# Refining the moose serum progesterone threshold to diagnose pregnancy

**DOI:** 10.1093/conphys/coad003

**Published:** 2023-02-19

**Authors:** Madeline Struck, William J Severud, Yvette M Chenaux-Ibrahim, Edmund J. Isaac, Janine L Brown, Seth A Moore, Tiffany M Wolf

**Affiliations:** Department of Veterinary Population Medicine, University of Minnesota, Saint Paul, MN 55108, USA; Department of Veterinary Population Medicine, University of Minnesota, Saint Paul, MN 55108, USA; Department of Biology and Environment, Grand Portage Band of Lake Superior Chippewa, Grand Portage, MN 55605, USA; Department of Biology and Environment, Grand Portage Band of Lake Superior Chippewa, Grand Portage, MN 55605, USA; Smithsonian Conservation Biology Institute, Center for Species Survival, National Zoological Park, Front Royal, VA, 22630, USA; Department of Biology and Environment, Grand Portage Band of Lake Superior Chippewa, Grand Portage, MN 55605, USA; Department of Veterinary Population Medicine, University of Minnesota, Saint Paul, MN 55108, USA

**Keywords:** serum progesterone, receiver operator characteristic curve, pregnancy, moose, * Alces alces*

## Abstract

Pregnancy determination is necessary for sound wildlife management and understanding population dynamics. Pregnancy rates are sensitive to environmental and physiological factors and may indicate the overall trajectory of a population. Pregnancy can be assessed through direct methods (rectal palpation, sonography) or indicated using hormonal assays (serum progesterone or pregnancy-specific protein B, fecal progestogen metabolites). A commonly used threshold of 2 ng/ml of progesterone in serum has been used by moose biologists to indicate pregnancy but has not been rigorously investigated. To refine this threshold, we examined the relationship between progesterone concentrations in serum samples and pregnancy in 87 moose (*Alces alces*; 64 female, 23 male) captured from 2010 to 2020 in the Grand Portage Indian Reservation in northeastern Minnesota, USA. Pregnancy was confirmed via rectal palpation (*n* = 25), necropsy (*n* = 2), calf observation (*n* = 25) or characteristic pre-calving behavior (*n* = 6), with a total of 58 females determined pregnant and 6 not pregnant; 23 males were included to increase the non-pregnant sample size. Using receiver operating characteristic analysis, we identified an optimal threshold of 1.115 ng/ml with a specificity of 0.97 (95% confidence interval [CI] = 0.90–1.00) and a sensitivity of 0.98 (95% CI = 0.95–1.00). Progesterone concentrations were significantly higher in cases of pregnant versus non-pregnant cows, but we did not detect a difference between single and twin births. We applied our newly refined threshold to calculate annual pregnancy rates for all female moose (*n* = 133) captured in Grand Portage from 2010 to 2021. Mean pregnancy rate during this period was 91% and ranged annually from 69.2 to 100%. Developing a reliable method for determining pregnancy status via serum progesterone analyses will allow wildlife managers to assess pregnancy rates of moose without devoting substantial time and resources to palpation and calf monitoring.

## Teaser

We refined the threshold of serum progesterone concentrations that indicated pregnancy in moose using samples from collared moose with known pregnancy status in Grand Portage Indian Reservation in northeastern Minnesota, USA. This refinement increased historical population-level pregnancy rates from an average of 81% to 91% of adult female moose.

The Grand Portage Band of Lake Superior Chippewa is a federally recognized Indian tribe in extreme northeastern Minnesota, USA, and proudly exercises its rights to food sovereignty through subsistence hunting and fishing. Moose are a primary subsistence food used by the Anishinaabeg (people) of Grand Portage Band historically and presently. Management for and research on maintaining this moose population as a vital subsistence species thus sets the context for this paper examining the pregnancy determination thresholds of serum progesterone of this culturally important resource.

## Introduction

Understanding population dynamics is needed for sound wildlife management. The relative importance of survival, reproduction and other vital rates can vary through time, for different species, and for disjointed populations (Johnson *et al.*, 2010). Decline in reproductive rates (pregnancy, litter size, age at first reproduction) can be an indicator of population trajectory (Eberhardt, 2002; Murray *et al.*, 2006; Lenarz *et al.*, 2010). Various factors can influence pregnancy rates (e.g. nutrition, adult sex ratios, stress), so understanding this reproductive parameter is important (Johnson *et al.*, 2010; Cook *et al.*, 2013; Morano *et al.*, 2013; Joly *et al.*, 2015; Newby and DeCesare, 2020).

Pregnancy can be assessed through direct methods (rectal palpation, sonography) or indicated using hormonal assays (serum progesterone or pregnancy-specific protein B, fecal progestogen metabolites) (Stewart *et al.*, 1985; Haigh *et al.*, 1993). Murray *et al.* (2006) reported slightly higher reliability using serum progesterone concentrations versus fecal assays to assess pregnancy in moose. A commonly used threshold of 2 ng/ml of progesterone in serum has been used by moose biologists to indicate pregnancy. This threshold was determined qualitatively based on a visual inspection of data trends and has not been otherwise investigated. In using this threshold in our own moose population research, we recognized several cases where female moose with serum progesterone concentrations below 2 ng/ml were in fact pregnant (as determined by rectal palpation of the fetus and subsequent calving events). Thus, the frequent use of this unvalidated threshold warrants the deriving of one quantitatively and with associated uncertainty (Haigh *et al.*, 1982; Testa and Adams, 1998; Murray *et al.*, 2006; Severud *et al.*, 2015; Wolf *et al.*, 2021).

The moose (*Alces alces*) population in northeastern Minnesota, USA, declined ~ 58% between 2006 and 2012, but between 2012 and 2020 had stabilized (ArchMiller *et al.*, 2018; DelGiudice and Giudice, 2022). Further, Minnesota has listed moose as a species of special concern due to the dramatic decrease in numbers in the northwestern and northeastern segments of the state-wide population (http://files.dnr.state.mn.us/natural_resources/ets/endlist.pdf). The northwestern population decline was accompanied by pregnancy rates averaging only 47.9% and low fecundity (twinning rate of 19%) (Murray *et al.*, 2006). Moose are an important subsistence species for tribal populations within and around the 1854 Treaty Ceded Territory in northeastern Minnesota, including the Grand Portage Band of Lake Superior Chippewa, Fond du Lac Band of Lake Superior Chippewa and the Bois Forte Band of Chippewa. The Grand Portage Band of Lake Superior Chippewa Department of Biology and Environment initiated a moose research program in 2010 to address concerns regarding declining moose population and maintaining subsistence harvest on the reservation. From 2010 to 2021, 147 unique adult moose have been captured, handled and collared over 190 captures.

Receiver operating characteristic (ROC) analysis is a method that can be used to better understand the relationship between a binary outcome and a continuous predictor for a diagnostic test (Zweig and Campbell, 1993; Mandrekar, 2010). Specifically, ROC analysis will identify an optimal threshold with the highest diagnostic sensitivity (probability that, in this case, a pregnant animal will test positive) and specificity (probability that a non-pregnant animal will test negative) in comparison to a “gold standard.” This method has been used in other species, such as West Indian manatees (*Trichechus manatus*) and dairy cows, to assess pregnancy (Faustini *et al.*, 2007; Tripp *et al.*, 2008; Newby and DeCesare, 2020).

Our goal was to elucidate the relationship between serum progesterone and pregnancy in free-ranging moose. Our specific objectives were to (1) quantitatively validate a serum progesterone concentration threshold indicative of pregnancy and (2) apply that threshold to archived serum progesterone data to estimate pregnancy rates of moose on and around the Grand Portage Indian Reservation (GPIR).

## Materials and Methods

### Study site

The Grand Portage Indian Reservation (227 km^2^) is bound to the north by Ontario, Canada, and on the west by federal, state and private lands of Minnesota, USA. Lake Superior borders the eastern and southern shores of the Reservation. Glacial activity produced a mix of ridges and valleys; elevation ranges from 183 to 553 m. There were 68 km of year-round streams and 89 km of intermittent streams. Seventeen inland water bodies covered 3.3 km^2^ within the Reservation boundaries, and there were 29 km^2^ of wetlands. The Reservation contained southern boreal forest species, with upland forests dominated by quaking aspen (*Populus tremuloides*), paper birch (*Betula papyrifera*), balsam fir (*Abies balsamea*), white spruce (*Picea glauca*) and jack pine (*Pinus banksiana*) (Minnesota Department of Natural Resources [MNDNR], 2015). Moose density was 0.27/km^2^, but their core range was inland away from Lake Superior (Oliveira-Santos *et al.*, 2021).

### Moose capture and handling

From 2010 to 2020, field crews captured and handled moose in accordance with requirements of the University of Minnesota Institutional Animal Care and Use Committee (protocols 1410-31945A, 1601-33318A, 1803-35736A and 1812-36635A) and guidelines of the American Society of Mammalogists (Sikes and the Animal Care and Use Committee of the American Society of Mammalogists, 2016). The earliest captures occurred on 21 January 2011 and the latest on 14 March 2017. Detailed capture methodology can be found in Wolf *et al.* (2021). Briefly, we captured adult moose on GPIR by helicopter through a commercial wildlife capture company as part of ongoing moose habitat and adult mortality studies conducted by the Grand Portage Band of Chippewa. We immobilized moose with either 10 mg thiafentanil oxalate, 4.5 mg carfentanil citrate or 8.5–10 mg etorphine hydrochloride (Wildlife Pharmaceuticals, Inc, Windsor, CO USA) in combination with 30–50 mg xylazine. We collected approximately 60 ml of blood from a jugular vein using a 16-ga needle attached to a 60-ml syringe. We transferred blood immediately into serum separator tubes (Corvace, Medtronic, Minneapolis, Minnesota, USA or BD Vacutainer, Becton, Dickinson and Company, Franklin Lakes, New Jersey, USA). We fitted cows with GPS Plus Iridium collars (Vectronic Aerospace GmbH, Berlin, Germany), equipped with a global positioning system (GPS) data-logger. From 2013 to 2019, an experienced veterinarian examined adult cows by rectal palpation for evidence of a fetus (Solberg *et al.*, 2003). Using a sterile, disposable vaginal speculum (Jorgensen Laboratories, Inc., Loveland, Colorado, USA), we implanted a vaginal implant transmitter (VIT; Vectronic Aerospace, Berlin, Germany) adjacent to the cervix in a subset of pregnant cows (Johnson *et al.*, 2006; Patterson *et al.*, 2013), which transmitted motion and body temperature data to the GPS collar at regular intervals. We administered naltrexone (20 mg/mg thiafentanil, 100 mg/mg carfentanil or 50 mg/mg etorphine) and 800 mg tolazoline (Arnemo *et al.*, 2003) intramuscularly to reverse anesthesia.

We monitored calving as previously described (Wolf *et al.*, 2021). Briefly, we monitored daily cow GPS location data that was transmitted via satellite every 30 minutes during 15 April–30 June each year (i.e. calving season) to enhance observation of movement behaviors associated with parturition. Parturition was signaled by a sharp drop in measured temperature from body to ambient environment and cessation of motion of the VIT following expulsion during calving. In females without a VIT implanted, we identified parturition by a significant increase in movement, followed by an abrupt geographical localization that remained constant over several days to weeks (McGraw *et al.*, 2014; Severud *et al.*, 2015). Calving was confirmed through calf capture and collaring, direct observation of the calf or calves or identification of calf tracks 48–72 hours post-parturition.

### Serum progesterone analysis

Within 2–3 hours of blood collection from adults, we centrifuged and extracted serum from separator tubes. Serum was refrigerated until it could be shipped overnight on ice to the endocrinology laboratory of the Smithsonian Conservation Biology Institute for progesterone concentration quantification.

Because of low mass recovery in accuracy tests for neat serum (y = 0.6302x—3.8343; R^2^ = 0.9996) or samples diluted 1:5 in assay buffer (Cat. No. X065, 5X, Arbor Assays, Ann Arbor, MI) (y = 0.4966x—2.6359; R^2^ = 0.9898), all samples were ether extracted. Serum (200–500 μl) was combined with ether at a 1:5 ratio (v/v) in glass tubes. Samples were vortexed at medium speed for 1 minute and then allowed to separate for 5 minutes. The tubes were placed in an −80°C freezer to solidify the serum layer, after which the ether was decanted into a second set of tubes and evaporated to dryness. The process was repeated, and the two ether supernatants were combined and dried under air. Extracted samples were reconstituted in assay buffer to the original volume of serum used for extraction, vortexed and sonicated.

Serum progesterone concentrations in extracted samples were analyzed using a double-antibody enzyme immunoassay (EIA) conducted in 96-well microtiter plates, pre-coated with secondary goat anti-mouse antibody (Cat. No. A008, Arbor Assays, Ann Arbor, Michigan, USA) diluted (10 μg/ml) in coating buffer (Cat. No. X108, 20X, Arbor Assays, Ann Arbor, Michigan, USA). To prepare the plates, secondary antibody was added to each well (150 μl) (Cat. No. 07–200-39, Fisher Scientific, Pittsburgh, Pennsylvania, USA), followed by incubation at room temperature (RT) for 15–24 hours. The contents of the wells were emptied, the plates were blotted dry, and blocking solution (Cat. No. X109, 10X, Arbor Assays, Ann Arbor, Michigan, USA) was added to each well (250 μl) and incubated for 15–24 hours at room temperature (RT). Contents of the wells were emptied and the plates blotted and dried at RT in a Dry Keeper (Sanplatecorp, Osaka, Japan) with loose desiccant in the bottom. After drying (humidity < 20%), plates were heat sealed in a foil bag with a 1-gram desiccant packet and stored at 4°C. At the time of use, plates were allowed to come to RT for ~ 30 minutes.

The EIA employed an anti-progesterone monoclonal primary antibody (CL425; 1:50000; C. J. Munro, University of California Davis, California, USA) and progesterone-3CMO-horseradish peroxidase (HRP; 1:110000) diluted in assay buffer (Cat. No. X065, 5X, Arbor Assays, Ann Arbor, Michigan, USA) and kept at 4^0^ C until use. Standards (Steraloids, Inc., Newport, Rhode Island, USA), internal controls and neat samples (50 μl each) were added to each well in duplicate, followed by addition of 25 μl HRP, and then 25 μl of primary antibody (except for non-specific binding wells). The assays were incubated at RT for 2 hours with shaking (500 rpm) before being washed five times with wash solution (Cat. No. X007, 20X, Arbor Assays, Ann Arbor, Michigan, USA) and blotted dry. High kinetic tetramethylbenzidine (TMB) (2.5 mmol/L, Prod. No. TMB-HK, Moss, Inc.; 100 μl) was added as the chromagen substrate to all wells. The assays were covered and incubated at RT without shaking for 30 minutes and then stopped with 50 μl of 1 N HCL. Plates were read immediately at 450 nm. The EIA was validated for moose ether-extracted serum by demonstrating parallelism between serially diluted extracts and the standard curve, and significant recovery of standard added to extracts before analysis (y = 0.9263x—0.0445 [R^2^ = 0.9981]). The assay sensitivity (based on 90% maximum binding) was 0.02 ng/ml; intra-assay and inter-assay coefficients of variation were < 10%.

### Statistical analyses

True pregnancy status of adult female moose (excluding yearlings) was assessed using a decision tree that ranked all the methods used in the field to confirm pregnancy and/or calving (Supplementary Fig. 1). Palpation was considered the gold standard for pregnancy determination (*n* = 25). If a fetus could not be detected on palpation or the cows were not palpated, we reviewed records to determine if there was later confirmation of calving. This included direct observation of a calf by Grand Portage biology staff within days of parturition (*n* = 25), as well as GPS documentation of the cow performing a characteristic pre-calving movement pattern (*n* = 6) (McGraw *et al.*, 2014; Severud *et al.*, 2015). A small number of moose were determined to be pregnant following identification of fetus(es) in utero at necropsy (*n* = 2). If a cow had no record of any of these observations, they were classified as non-pregnant (*n* = 6). Male moose with serum progesterone data were included as non-pregnant (or negative) controls (*n* = 23). We included males to yield more non-pregnant data because of the limited number of non-pregnant female moose in our study sample. A t-test was utilized to determine if the serum progesterone levels were significantly different between the males and non-pregnant females. In total, 58 pregnant and 29 non-pregnant moose were used in the analysis.

**Figure 1:**
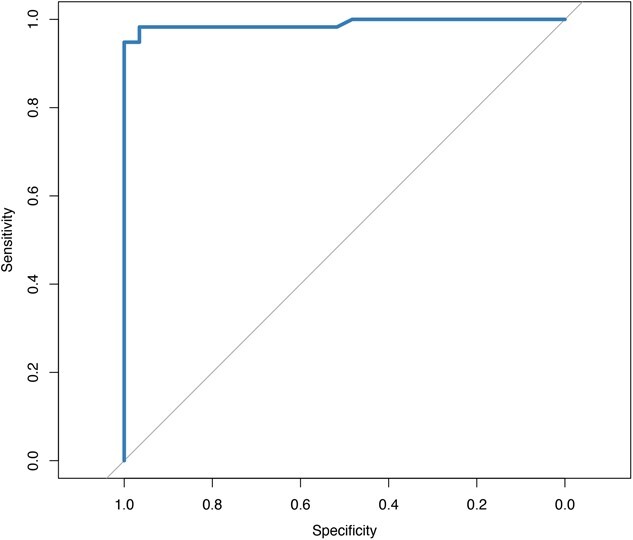
Receiver operating characteristic curve of sensitivity (probability of detecting a true positive) and specificity (probability of detecting a true negative) using serum progesterone concentrations of pregnant and non-pregnant moose from Grand Portage Indian Reservation, northeastern Minnesota, USA, 2010–2020 (*n* = 87).

We used a receiver operating characteristic (ROC) curve to calculate a progesterone threshold with the highest specificity and sensitivity to diagnose pregnancy (pROC package in R) (Robin *et al.*, 2011; R Core Team, 2020). The area under the curve (AUC) was computed from the ROC curve to assess the strength of the tool’s predictive power. We next examined the relationship between fecundity (number of calves per pregnancy) and serum progesterone concentration. Raw progesterone data from pregnant and non-pregnant female moose with known numbers of calves (*n* = 61) were analyzed by a D’Agostino test for normality. The sample data were not normally distributed ($ \alpha$ = 0.05), so it was logarithmically transformed. An analysis of variance (ANOVA) with Tukey’s honestly significant difference (HSD) post hoc test assessed pairwise differences between the progesterone concentration of moose that were not pregnant (*n* = 6), had a singleton birth (*n* = 37) or had twins (*n* = 18; $ \alpha$ = 0.05). We then used our optimized threshold from the ROC analysis to estimate pregnancy rates of all 126 captured and handled female moose. Raw data and code for all analyses are available at the Data Repository for University of Minnesota (DRUM) (https://hdl.handle.net/11299/226884).

## Results

We analyzed blood serum from 173 moose captured, handled and collared from 2010 to 2020 before narrowing our sample down to the final 87 with comparative pregnancy data for ROC analysis (64 females, 23 males). Mean serum progesterone concentration was 2.561 ng/ml (standard error: 0.220, range = 0.07–10.76). The t-test revealed that there was not a significant difference between P4 levels in male and non-pregnant moose (p > 0.05). The ROC analysis determined an optimal serum progesterone concentration of 1.115 ng/ml with a specificity of 0.97 (95% confidence interval [CI] = 0.90–1.00) and a sensitivity of 0.98 (95% CI = 0.95–1.00). The AUC of the curve was 0.99 (Fig. 1). We identified alternate thresholds that maximized specificity or sensitivity (Supplementary Table S1).

**Figure 2:**
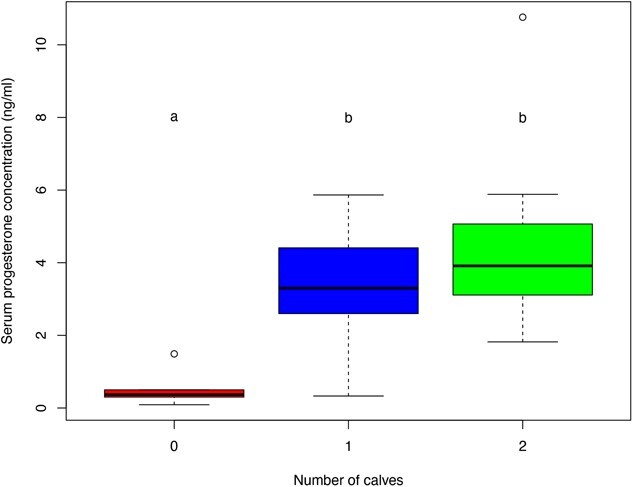
Boxplot comparing serum progesterone concentrations (ng/ml) among female moose that gave birth to 0, 1 or 2 calves, Grand Portage Indian Reservation, northeastern Minnesota, USA, 2010–2020 (*n* = 61). Boxes depict interquartile range, dark lines are median values and whiskers are 1.5× interquartile range. Different letters indicate significant pairwise differences (*P* < 0.05).

**Figure 3:**
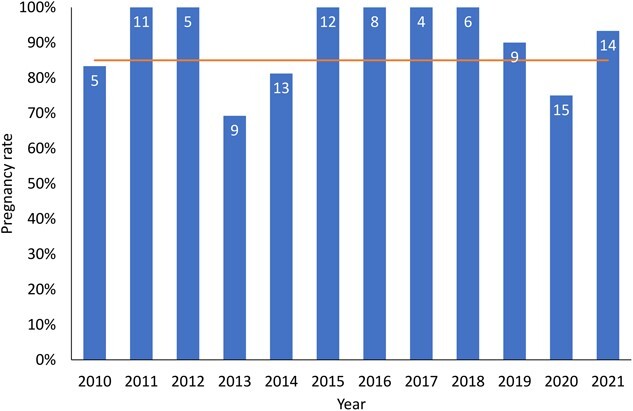
Pregnancy rates of adult female moose (*A. alces*) in Grand Portage Indian Reservation, northeastern Minnesota, USA, as estimated using a serum progesterone concentration threshold of 1.115 ng/ml. Number in bars represent number of pregnant moose each year, horizontal line represents the North American mean pregnancy rate (Ruprecht *et al.,* 2016).

The overall ANOVA indicated differences in serum progesterone concentrations among cows with different numbers of offspring (F_2,58_ = 32.4, *P* < 0.001). A Tukey’s HSD post-hoc pairwise comparison test revealed progesterone concentrations of cows that were not pregnant were lower than cows that birthed a single or twin calves (*P* < 0.001; Fig. 2), but concentrations of cows that birthed a single or twin calves were not different (*P* = 0.17). Annual pregnancy rates for adult female moose captured in Grand Portage ranged from 69.2 to 100%, with a mean of 91% (Fig. 3).

## Discussion

Our ROC model established the first refined, quantitatively derived threshold for serum progesterone concentration to diagnose pregnancy in moose. The high AUC, in combination with a high sensitivity and specificity, indicates that a threshold of 1.115 ng/ml has strong predictive value and could have practical use to wildlife managers. When applied to the retrospective moose data from Grand Portage, the mean pregnancy rate increased to 91% compared to the 81.2% pregnancy rate determined by the previously used 2 ng/ml threshold. Of our sample of 64 adult female moose with comparable pregnancy data, 8 with confirmed calves would have been falsely identified as not pregnant with the 2 ng/ml threshold. We reported weak evidence for discerning single from multiple births, and low sample numbers may have contributed to this. Pregnancy-specific protein B (PSPB) does increase with the number of fetuses in other mammals (Willard *et al.*, 1995), and has been used in elk (*Cervus elaphus*) and moose as well (Huang *et al.*, 2000).

Other serum progesterone thresholds have been utilized in moose studies without rigorous statistical backing. For example, 1.26 ng/ml was chosen as a cutoff by Milner *et al.* (2013), as 66 out of 79 moose captured with progesterone above that level were determined pregnant while no moose with progesterone below were pregnant. In another study, moose that were not pregnant demonstrated progesterone concentrations < 0.2 ng/ml, whereas all other moose that were pregnant ranged 1.8–4.7 ng/ml. Interestingly, in one case, a cow with extensive uterine and ovarian adhesions that likely prevented pregnancy demonstrated a progesterone level that fell within the observed range of pregnant cows (Testa and Adams, 1998). Reproductive pathology could influence serum progesterone concentrations that may affect pregnancy interpretation. While there may be some concerns about validity of results produced by different laboratories (Gail *et al.*, 1996), our methods are robust to these concerns. Specifically, we used a standard assay validation technique, with parallelism and recovery tests, that ensure repeatability of results across labs.

It is inherently difficult to determine the pregnancy status of a free-ranging animal. Rectal palpation is invasive and requires an experienced practitioner (Solberg *et al.*, 2003), while ultrasound equipment is costly and cumbersome for field use (Duquette *et al.*, 2012). Follow-up observations of neonates after parturition can cause stress to the cow and be time-consuming for field personnel (DelGiudice *et al.*, 2018). A blood sample is needed to evaluate pregnancy using serum progesterone concentrations, but blood collection is a routine part of most animal handling operations. Provided moose captures occur during the prolonged period of elevated progesterone during gestation (Stewart *et al.*, 1985), serum progesterone from blood drawn at captures can yield valuable reproductive information. In pregnant captive moose, serum progesterone concentrations began to decline 40–50 days prepartum (Stewart *et al.*, 1985). We captured and sampled moose in January–March and, as calves were born in May–June, we are confident that in this study we sampled animals during this window of elevated progesterone levels.

We utilized a variety of field observations to inform our gold standard of comparison for pregnancy diagnosis, although it is important to recognize the potential for misclassification bias associated with this approach. For example, a cow pregnant at capture but not confirmed via palpation, characteristic pre-parturition movements or direct observation of a calf following parturition (possibly due to abortion or stillbirth) may be misclassified as not pregnant. There are rare occasions where cow moose have been observed to calve without any observable localization (Severud *et al.*, 2015). Although our team has not documented instances of abortion in this study population, it has been observed elsewhere in moose (particularly in association with rectal palpation for pregnancy) (Solberg *et al.*, 2003), and we have observed stillbirths in this population (Wolf *et al.*, 2021). In these cases, we would expect the serum progesterone concentration to be elevated because of pregnancy, but lack of a confirmed calf because of abortion or stillbirth might result in a classification of non-pregnant (particularly if palpation data were not available). Thus, this kind of misclassification of true pregnancy status could result in a ROC-derived threshold biased at a higher level. It is also difficult to determine the number of calves born to a cow depending on how short the window is between birth and visualization. Longer windows leave more room for predation and other causes of calf loss that could lead to misclassification of a twin pregnancy as a singleton (Severud *et al.*, 2019; Wolf *et al.*, 2021). This may have contributed to the lack of a statistically significant difference between the progesterone concentrations of moose with singleton and twin pregnancies.

Due to few non-pregnant female moose, male moose were included in the non-pregnant category. Studies suggest that the P4 levels in males and non-pregnant females are similar. For example, nulliparous female and male humpback whales (*Megaptera novaeangliae*) both had lower progesterone concentrations in baleen compared to reproductively active females, even during the interpregnancy intervals (Lowe *et al.*, 2021). For moose, nulliparous females are likely yearlings and two-year-olds (Schwartz, 2007). Any moose identified as a yearling was excluded from evaluation, but there is still the potential for incorrect aging at handling. Whether or not a significant difference is present between male and non-pregnant, multiparous female P4 levels in moose has not been rigorously tested. Given the high proportion of males to non-pregnant females in the non-pregnant category of this study, this is a potential source of bias that could artificially lower the threshold. However, there was not a significant difference between the P4 levels in males and non-pregnant females in this study.

Studies attempting to determine optimal serum progesterone thresholds to diagnose pregnancy using ROC curves have been performed in both West Indian manatees and dairy cattle. In manatees, 0.40 ng/ml was the chosen cutoff with a specificity of 86.7% and a sensitivity of 93.3% (Tripp *et al.*, 2008). This value was selected as it maximized both the sensitivity and specificity. In dairy cattle, the threshold was assessed using the progesterone concentrations in whey rather than serum. In this case, the cutoff was 0.26 ng/ml with a specificity of 70.9% and a sensitivity of 98.2%. Sensitivity was intentionally maximized in this study as the economic cost to producers of a false negative cow is much higher than that of a false positive cow (Faustini *et al.*, 2007). We provide alternate thresholds so practitioners can either maximize sensitivity or specificity to suit their specific project goals (Supplementary Table S1).

The revised mean annual pregnancy rate of 91% places the Grand Portage moose population within the range of rates seen in North American moose populations established by Ruprecht *et al.* (2016). A meta-analysis comparing multiple methods of pregnancy determination in North American moose populations (fetal count, fecal progesterone, serum progesterone, rectal palpation, PSPB) reported rates ranging from 69 to 100%, with a mean value of 85.0% (±1.3% SE). One of the methods employed, a serum assay of PSPB, may be a more robust indicator of fecundity than serum progesterone as its concentration increases with placental mass (Ruprecht *et al.,* 2016).

Moose populations in Minnesota and across North America, while currently stable, have experienced drastic declines in recent decades (Lenarz, 2007; Timmermann and Rodgers, 2017; DelGiudice, 2020). This is especially concerning given that moose are integral to the food security of indigenous populations throughout the region (Priadka *et al.*, 2022). It is important for wildlife managers to be able to gather data on population dynamics such as birth rates to monitor for any further declines or changes to the population; integrated population models can leverage these types of data to produce more precise and accurate population projections (Severud *et al.*, 2022). Knowing the threshold of serum progesterone to diagnose pregnancy in moose will allow managers to gather that information without time-intensive and stressful follow-up captures. With this data in hand, managers will be better able to assess when action is needed to ensure persistence of the population.

## Supplementary Material

Web_Material_coad003

## Data Availability

Raw data and code for all analyses are available at the Data Repository for University of Minnesota (DRUM) (https://hdl.handle.net/11299/226884).
