# Supplementary material for: Refining the moose serum progesterone threshold to diagnose pregnancy
Source: Conserv Physiol. 2023 Feb 19;11(1):coad003. doi: 10.1093/conphys/coad003 (PMC10660365; doi:10.1093/conphys/coad003)
Supplement: Web_Material_coad003 [file web_material_coad003.pdf]

Supplemental Materials

Supplemental Table 1. Serum progesterone (P4) concentration (ng/ml) thresholds and associated sensitivity, specificity, positive predictive value, and negative predictive value for detecting pregnancy in free-ranging moose in Grand Portage Indian Reservation, northeastern Minnesota, USA, 2010–2020 (n = 87).

P4 threshold (ng/ml)	Sensitivity	Specificity	Positive predictive value	Negative predictive value
0.320	1.00	0.48	0.79	1.00
**1.115**	**0.98**	**0.97**	**0.98**	**0.97**
1.545	0.95	1.00	0.95	0.91

Supplemental Figure Legends

Supplemental Figure 1. Decision tree for determination of pregnancy status of moose from Grand Portage Indian Reservation, northeastern Minnesota, USA, 2010–2020. White rounded rectangles indicate different decision methods used for determination. Green diamonds depict the number of females found pregnant with each method, and red diamonds depict those found not pregnant.

Supplemental Figure 2. Density plot indicating serum progesterone concentration (ng/ml) counts of pregnant (blue dashed line) and non-pregnant (solid black line) moose from Grand Portage Indian Reservation, northeastern Minnesota, USA, 2010–2020. The vertical red line indicates the threshold we identified using receiver operating characteristic analysis.

Supplemental Figure 3. Density plot indicating serum progesterone concentration (ng/ml) counts of cows that gave birth to 1 calf (solid black line) or 2 calves (blue dashed line) from Grand Portage Indian Reservation, northeastern Minnesota, USA, 2010–2020. The vertical red line indicates the threshold we identified using receiver operating characteristic analysis.

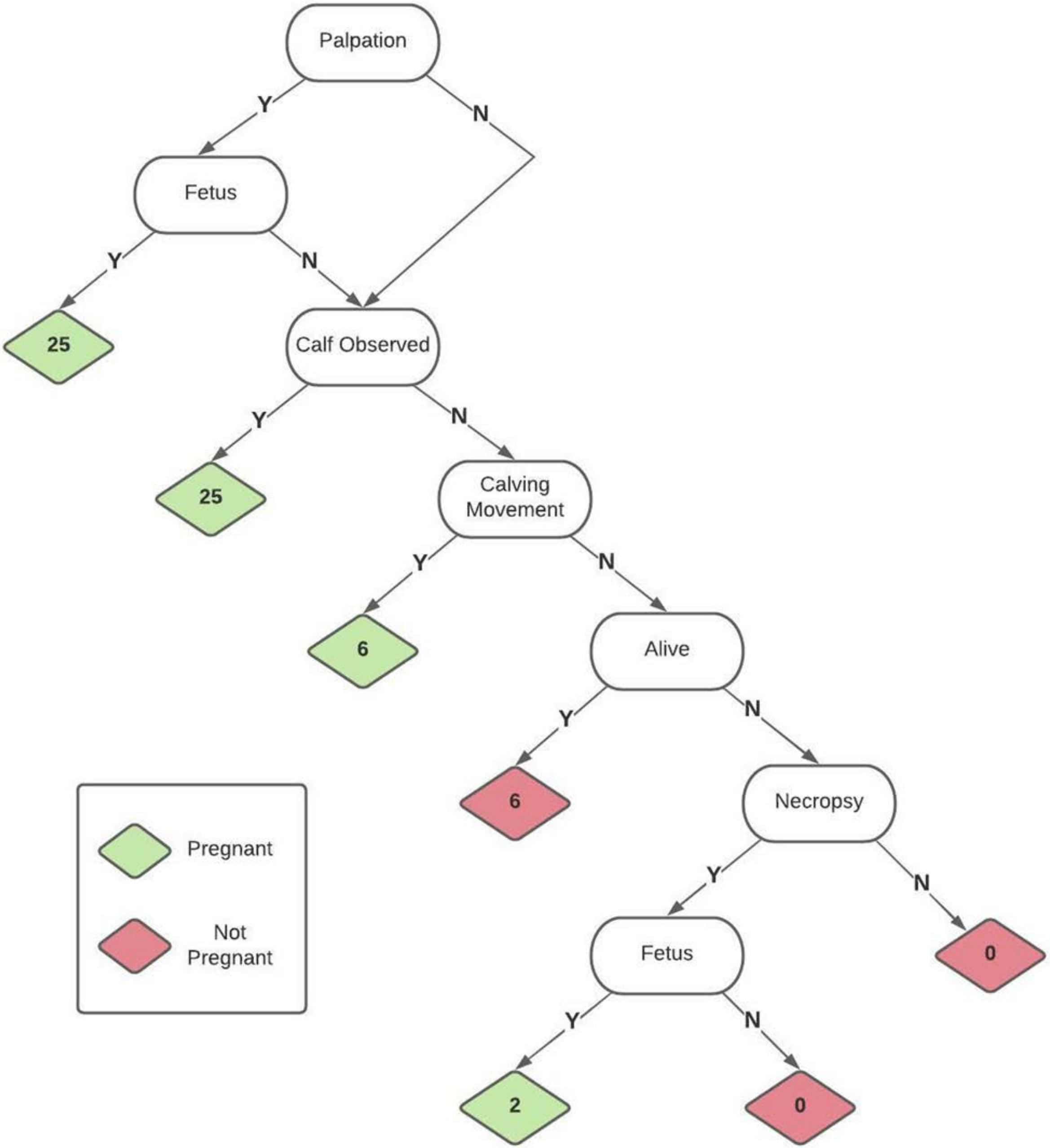

Palpation
Y
N
Fetus
Y
N
25
Calf Observed
Y
N
25
Calving Movement
Y
N
6
Alive
Y
N
6
Necropsy
Y
N
Fetus
Y
N
0
2
0
Pregnant
Not Pregnant

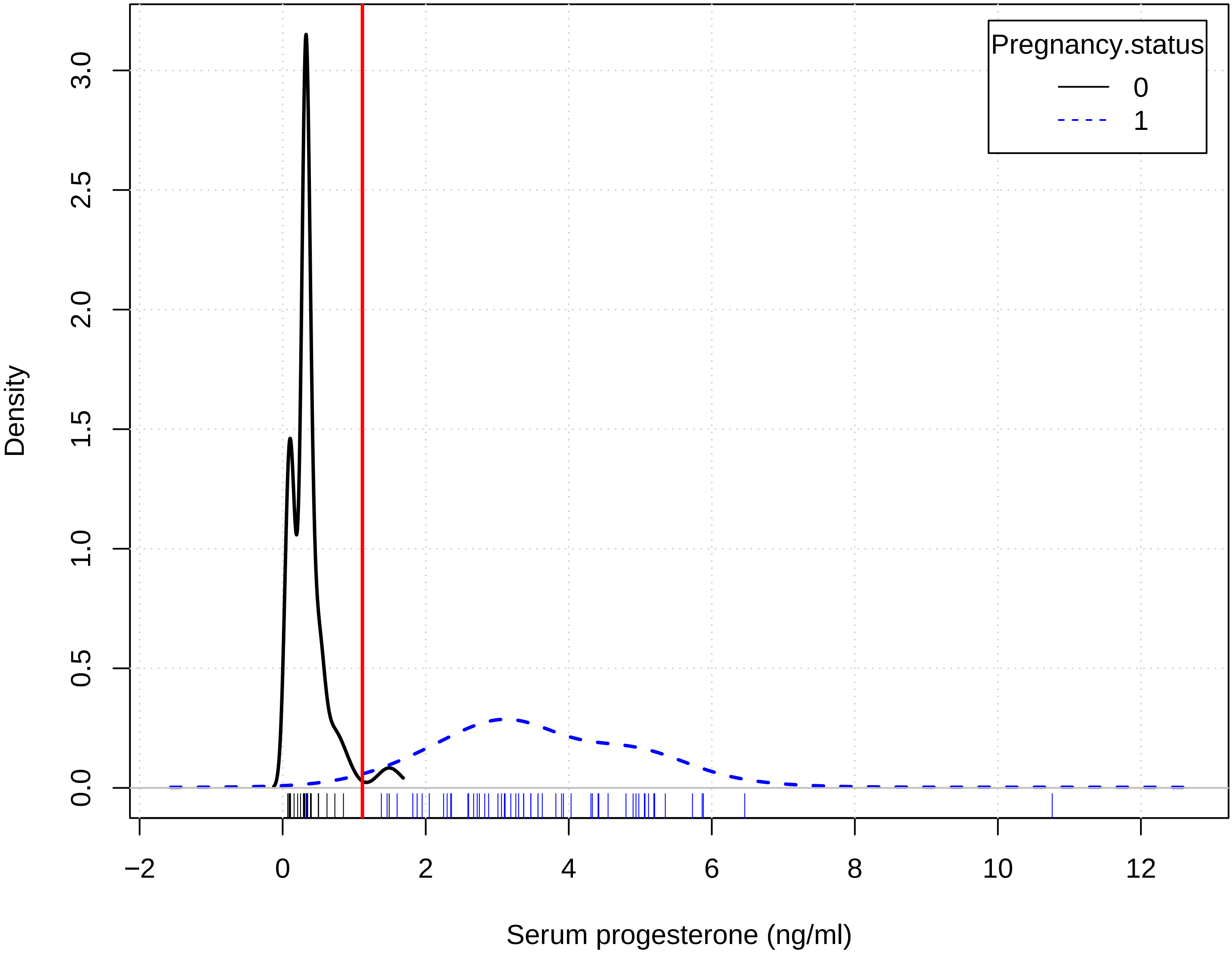
Pregnancy.status
0
1
Density
0.0
0.5
1.0
1.5
2.0
2.5
3.0
−2
0
2
4
6
8
10
12
Serum progesterone (ng/ml)

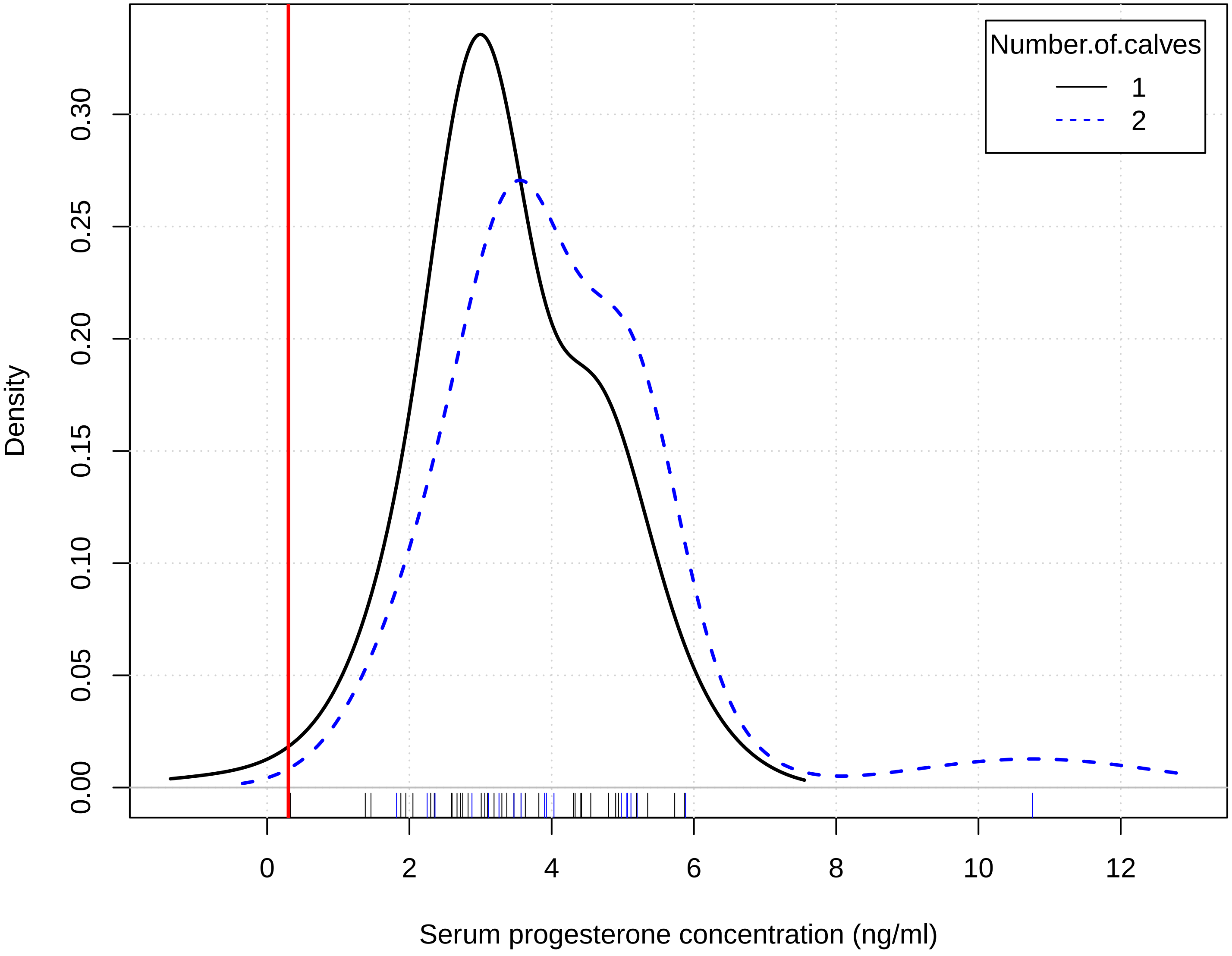
Number.of.calves
1
2
Density
0.00
0.05
0.10
0.15
0.20
0.25
0.30
0
2
4
6
8
10
12
Serum progesterone concentration (ng/ml)
